# The fundal cerebrospinal fluid cap in vestibular schwannoma surgery: a predictor of outcome after the retrosigmoid approach

**DOI:** 10.1007/s10143-026-04305-x

**Published:** 2026-05-02

**Authors:** Ryan Beerling Dolovac, Leon Lai, Jordan Jones, Christopher Ovenden, Jeremy Kam, Gina Arena, Mendel Castle-Kirszbaum

**Affiliations:** 1 School of Medicine, University of Western Australia, Perth, WA Australia; 2 Department of Neurosurgery, Monash Health, Melbourne, VIC Australia; 3 Department of Surgery, Monash University, Melbourne, VIC Australia; 4 Department of Neurosurgery, Royal Melbourne Hospital, Melbourne, VIC Australia; 5 Department of Neurosurgery, Royal Adelaide Hospital, Adelaide, Australia; 6 Faculty of Health and Medical Sciences, Adelaide Medical School, Adelaide, SA Australia

**Keywords:** Fundal cap, Vestibular schwannoma, Facial nerve, Hearing, Acoustic neuroma, CSF cap

## Abstract

The fundal cerebrospinal fluid (CSF) cap is a radiological finding correlating to a pocket of CSF lateral to vestibular schwannomas in the fundus of the internal acoustic meatus. Its presence may increase the likelihood of good facial nerve outcome and hearing preservation after microsurgical resection. A systematic review of the literature was performed. Studies that reported the association of a fundal fluid cap with postoperative outcomes including facial nerve outcome, hearing preservation and extent of resection were included. A total of 17 studies were included, comprising 2370 patients. Studies were generally at high risk of bias. The presence of a fundal cap was associated with significantly higher rate of good (HB I-II) facial nerve outcome after retrosigmoid approaches (OR 6.04; 95%CI 2.79–13.11), but not after translabyrinthine and middle fossa approaches. A fundal cap was associated with an increased rate of gross total resection (OR 2.13; CI: 1.51–3.00) and hearing preservation after retrosigmoid (OR 3.37; 95% CI: 2.32–4.90), but not middle fossa approaches (OR 1.47; 95% CI: 0.89–2.44). A fundal cap was also predictive of hearing preservation after radiosurgery. The fundal CSF cap is an important predictor of facial nerve function and hearing preservation after retrosigmoid craniotomy for vestibular schwannoma. Its importance in middle fossa and translabyrinthine surgery is less clear, which reflects the anatomical considerations of each approach. The presence or absence of a fundal cap should be documented preoperatively and used to guide more nuanced risk assessment for preoperative patient counselling.

## Introduction

Interventions for vestibular schwannoma aim to control tumour growth and maximise extent of resection while preserving facial nerve function and hearing. Several patient and tumour characteristics have been associated with these outcomes, including age, tumour size, tumour consistency, surgical approach, and the presence of NF2-related schwannomatosis [1–3].

Beyond tumour size, radiological predictors including the fundal cerebrospinal fluid (CSF) cap, have been demonstrated to correlate with facial nerve outcome and hearing preservation after microsurgical resection [4, 5]. The fundal cap is a volume of high T2 signal in the lateral aspect of the internal auditory meatus on MRI. Anatomically, this represents a pocket of CSF within the fundus of the meatus, lateral to the tumour. Vestibular schwannoma grow within the arachnoid pocket of the vestibular nerves, preserving an arachnoid plane medially on the outer arachnoid wall of the cerebellopontine angle cistern, and a plane laterally within the meatal arachnoid [6]. The fundal cap CSF space defines the lateral aspect of the tumour arachnoid pocket, providing an undisturbed starting point to define the perineurial dissection plane off the facial and cochlear nerves from lateral to medial [7].

Accurate prediction of surgical outcomes is crucial to form informed decision making in vestibular schwannoma as in many cases there is equipoise between surgical, conservative, and radiosurgical management [8]. Reliable pre-operative assessment of rates of extent of resection, hearing loss, and facial nerve perseveration would provide much needed clarity when counselling patients preoperatively and facilitate formulation of realistic expectations for patients, their families, and their surgeons [9]. In this systematic review, we evaluate the existing literature on the fundal CSF cap to determine its predictive value for extent of resection, hearing outcomes, and facial nerve preservation in vestibular schwannoma surgery.

## Methods

A systematic search of the literature was conducted using the PubMed, Embase, Cochrane Library, and Scopus databases in accordance with the PRISMA statement. All studies from database inception until 25th March 2025 were queried using the search string:

Fundal cap OR CSF cap OR Cerebrospinal fluid cap OR Fluid cap) AND (Vestibular schwannoma OR acoustic neuroma OR acoustic neurinoma).

Inclusion criteria were: (1) cohort studies or large case series; (2) preoperative measurement of a fundal CSF cap on MRI; (3) clinical outcome including facial nerve function, hearing preservation, or extent of resection stratified by fundal cap. Tumour size, presence of NF2-related schwannomatosis, and surgical approach were recorded but not used as screening criteria.

Exclusion criteria included conference abstracts, case reports, studies published in languages other than English, studies that did not report clinical outcomes stratified by fundal cap presence, and studies that republished the same patient database. The references of included studies were also individually hand-searched to identify additional eligible studies.

Included studies underwent independent, blinded, data extraction including study year, study size, surgical technique, and fundal CSF cap presence, extent of resection, facial nerve outcome, and hearing outcome by two reviewers (RBD, MCK). Facial nerve outcome was universally reported using the House-Brackmann (HB) grade, with good facial nerve function defined as HB grades I and II, and poor facial nerve outcome as HB grades III-VI. Hearing was reported by the American Academy of Otolaryngology–Head and Neck Surgery (AAO) classification system, with serviceable hearing defined as AAO Class A or B. Bias was assessed using the ROBINS-I-V2 risk of bias assessment scale for non-randomised studies.

Extracted data from studies was tabulated and key results summarised in prose. Studies that presented their results in extractable binary endpoints underwent meta-analysis with the common effects model and unadjusted odds ratios reported. Interstudy heterogeneity was measured with the Cochran’s Q test. An alpha < 0.05 was defined as statistically significant. Publication bias was primarily evaluated through the construction of funnel plots for each outcome, complemented by statistical tests where applicable. Sensitivity analysis was performed using leave-one-out analysis to determine the influence of individual studies on the overall effect estimates. Analysis was performed using R 4.4.2.

## Results

### Included studies

The literature search identified 59 studies, of which a total of 17 studies were included in this review. Studies were overall of a high risk of bias, and comprised a total of 2370 patients (Table 1) [2, 4, 5, 10–24]. Where data were available, the mean age was 50.2 years, with a relatively equal spread of male (*n* = 1127) and female (*n* = 1243) patients. The mean tumour greatest dimension ranged from 7.5 mm to 36 mm, with a relatively equal spread of tumour lateralisation (403 left- and 358 right-sided). The included studies utilized retrosigmoid (*n* = 1349), translabyrinthine (*n* = 297), middle fossa (*n* = 459) approaches, as well as radiosurgery (*n* = 233).

**Table 1:** Summary of studies assessing the effect of fundal CSF cap on outcomes in vestibular schwannoma

Study (Year)	*N*	Fundal CSF Cap (Present/Total)	Fundal Cap Definition	Surgical Approach	Mean/Median tumour Size (mm)
Macielak [22]	502	336/502	Absent/Present	338 Retrosigmoid + 164 Translabyrinthine	18
Fujita [10]	143	102/143	Absent/Present	Retrosigmoid	NR
Hildrew [12]	127	NR	Absent/Present	Radiosurgery	NR
Van Rompaey [5]	110	46/110	Absent/Present	42 Retrosigmoid + 68 Translabyrinthine	18.4
Bojrab [19, 25]	106	48/106	Absent/Present	Radiosurgery	NR
Shi [16]	185	79/185	Absent/Present	Retrosigmoid	24
Sun [18]	138	138/138	Fundal Cap Distance	Middle Fossa	9.7
Kosty [13]	63	19/48	Absent/Present	Middle Fossa	10
Raheja [15]	78	NR	Absent/Present + Fundal Cap Distance	Middle Fossa	7.5
Zhou [20]	441	198/404	Absent/Present	Retrosigmoid	29
La Monte [14]	63	NR	Absent/Present	Middle Fossa	9.1
Kobayashi [21]	45	NR	Fundal Cap Distance	Middle Fossa	11.3
Sullivan [17]	36	18/35	Fundal Cap Distance	20 Translabyrinthine + 16 Middle Fossa	NR
Somers [4]	26	20/26	Absent/Present	Retrosigmoid	NR
Goddard [24]	101	76/101	Absent/Present	Middle Fossa	10
Mohr [22]	63	36/63	Absent/Present	Retrosigmoid	NR
Di Maio [23]	28	15/26	Absent/Present	Retrosigmoid	36

*EOR* Extent of Resection, *HO* Hearing Outcomes, *FNO* Facial Nerve Outcome, *NR* Not reported

Bias assessment was performed using the ROBINS-I-V2. Studies were primarily retrospective cohort studies, with 12 studies rated as moderate risk of bias and 5 studies carrying a severe risk of bias. All studies were rated low risk in the domains of participant selection, adherence to intended interventions, and outcome measurements. However, all studies were at moderate risk of bias due to confounding factors, with approximately 60% of papers at risk of bias due to intervention classification.

### Measurement of fundal cap

**Fig. 1:**
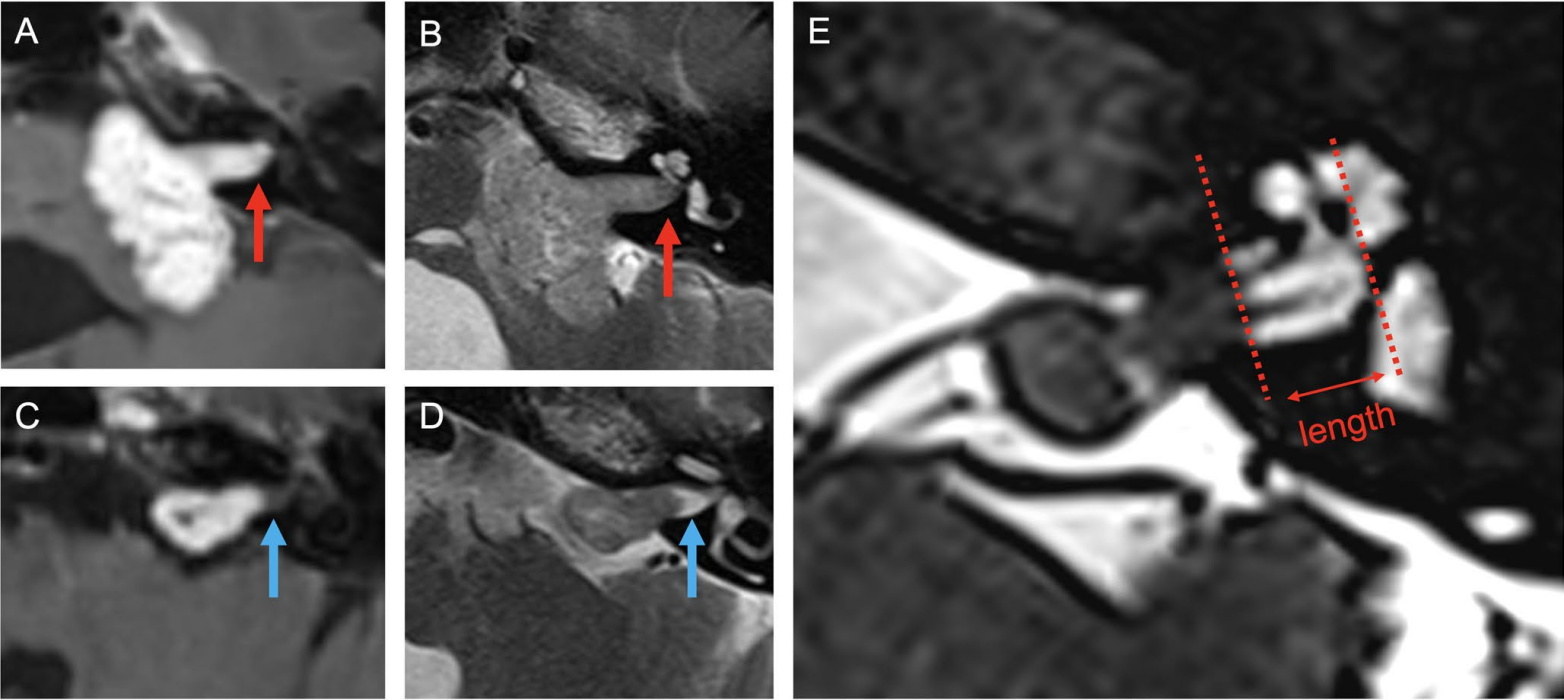
MRI examples of a fundal CSF cap. T1 Post-contrast (**A**) and T2 (**B**) axial MRI acquisitions of a patient with NF2 related schwannomatosis with a large left sided vestibular schwannoma. Contrast enhancing tumour is seen all the way to the fundus of the meatus, and there is an absence of high T2 signal (indicating the absence of cerebrospinal fluid) in the fundus of the meatus, suggesting no fundal CSF cap (red arrows). T1 Post-contrast (**C**) and T2 (**D**) axial MRI acquisitions of a patient with a sporadic left sided vestibular schwannoma demonstrating a clear separation between the contrast enhancing tumour and the meatal fundus, with high T2 signal in the fundus in keeping with a fundal cap (blue arrows). Fine slice heavily T2 weighted imaging of the same patient (**E**) can be used to measure the length of this cap

Where data were available, the presence of a fundal CSF cap was reported in 1131 of 1887 patients (59.9%). Fundal caps were predominantly defined in a binary fashion (presence vs. absence) (77.8%, 14/17). In 4 studies, the fundal cap depth (length) was measured as per the technique in Fig. 1. Mean fundal cap length ranged from 0.76 mm to 3 mm.

### Facial nerve outcome

Seven studies reported facial nerve outcomes in relation to fundal cap presence (Table 2) [2, 5, 10, 11, 16, 17, 20, 21].

**Table 2:** Summary of studies assessing facial nerve outcome

Study (Year)	*N*	Surgical Approach	Outcome
Fujita [10]	143	Retrosigmoid	Fundal cap associated with good CN7 outcome (HB1-2) at 24mo, when tumour size and intraoperative neuromonitoring response were accounted for (*p* = 0.03). At 24mo, good CN7 outcome was seen in 97% of fundal cap cases vs. 82.9% without.
Macielak [22]	502	338 Retrosigmoid + 164 Translabyrinthine	Fundal cap not significantly associated with a poor facial nerve outcome (HB grade 3+)
Shi [16]	185	Retrosigmoid	Good CN7 outcome (HB1-2) was seen in 92.4% of patient with fundal cap vs. 67.9% of those without (*p* = 0.007).
Van Rompaey [5]	110	42 Retrosigmoid + 68 Translabyrinthine	At 1 month postoperatively, good CN7 outcome (HB1-2) was seen in 87.0% of patients with fundal cap vs. 70.3% of those without. At 1 year postoperatively, good CN7 outcome (HB1-2) was seen in 89.1% of patients with fundal cap vs. 81.3% of those without.
Kobayashi [21]	45	Middle Fossa	No association between fundal cap length and CN7 outcomes
Sullivan [17]	36	20 Translabyrinthine + 16 Middle Fossa	Fundal cap associated with poorer CN7 outcome.
Zhou [20]	441	Retrosigmoid	Patients with complete obliteration of CSF signal in the meatus had worse CN7 outcome than those with 75–99% obliteration (HB1-2 52% vs. 73.9%, *p* < 0.001)

*HB* House-Brackman grade

**Fig. 2:**
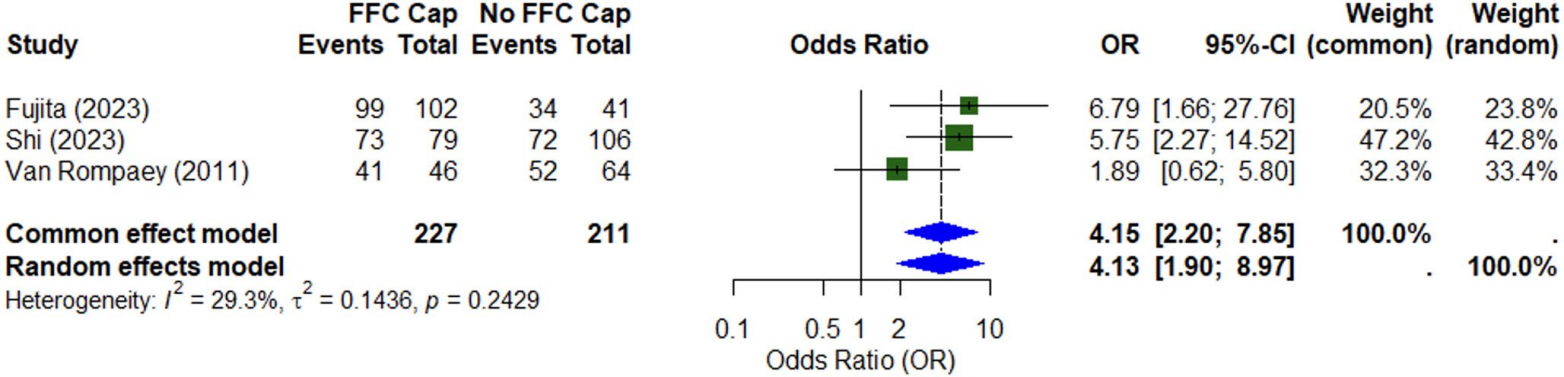
Meta-analysis of studies assessing the effect of a fundal cap on good facial nerve outcome after vestibular schwannoma surgery

Three studies reported rates of good facial nerve outcome (HB I-II) categorised by the presence or absence of a fundal cap. Two studies utilised the retrosigmoid approach alone while the other was a mixed retrosigmoid and translabyrinthine cohort [5, 10, 16]. Meta-analysis of these three studies (*n* = 438) demonstrated a four-fold greater likelihood of good facial nerve outcome in patients with a fundal CSF cap (OR 4.15; 95% CI: 2.20–7.85) (Fig. 2). When only the pure retrosigmoid cohorts were included there remained a strong correlation with good facial nerve outcome in patients with a fundal CSF cap (OR 6.04; 95% CI 2.79–13.11) [10, 16]. The association of a fundal cap and good facial outcome was independent of tumour size, age, or position relative to the long-axis of the internal acoustic meatus [10, 11, 16].

In a large (*n* = 603) mixed cohort of retrosigmoid and translabyrinthine approaches, no independent relationship between fundal cap and facial outcome was seen after tumour size was accounted for, but subgroup analysis by approach was not performed [2]. In a smaller mixed cohort of retrosigmoid and translabyrinthine surgery (*n* = 110), the absence of a fundal cap was associated with more than twice the risk of poor facial nerve outcome (HB III-VI) (29.7% vs. 13.0%, *p* = 0.04).

In a cohort of 45 patients who underwent middle fossa approach for small vestibular schwannomas, the length of the fundal cap was not associated with facial nerve outcomes at three months post-operatively [21]. However, in a combined cohort of patients treated via middle fossa and translabyrinthine approaches, a longer fundal cap was linked to poorer facial nerve outcomes [17]. Patients with longer fundal caps also tended to have larger tumours, which may confound these findings.

No publication bias was evident on inspection of constructed funnel plots. Linear regression test of funnel plot asymmetry was not significant (t = − 0.01,df = 1,*p* = 0.9950), suggesting no evidence of publication bias. Leave-one-out analysis did not reveal any single study disproportionately influencing the overall outcome.

### Extent of resection

**Fig. 3:**
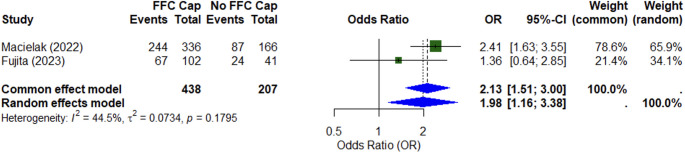
Meta-analysis of studies assessing the effect of a fundal cap on extent of resection after vestibular schwannoma surgery

Two studies reported on the relationship between extent of resection (EOR) and a fundal cap (Table 3) [2, 10]. After retrosigmoid craniotomy, extent of resection was not associated with a fundal cap, while in a mixed cohort of retrosigmoid and translabyrinthine approaches, the presence of a fundal cap was associated with greater extent of resection, independent of tumour size and patient age [2, 10]. Meta-analysis of these two studies, comprising 645 patients, demonstrated over twice the likelihood of gross total resection when a fundal fluid cap was present (OR 2.13; CI: 1.51–3.00) (Fig. 3). The surgical strategy for Fujita et al. was to prioritise facial nerve function over gross total resection, with a resulting gross total resection rate of 63.6%, while Macielak et al. did not explicitly comment on surgical philosophy in their manuscript [2, 10].

**Table 3:** Summary of studies assessing extent of resection

Study (Year)	*N*	Surgical Approach	Definition of EOR	Outcome
Macielak [22]	502	338 Retrosigmoid + 164 Translabyrinthine	GTR = No residual at surgery and no residual on MRI NTR = Residual < 5 × 5 × 2 mm at surgery and no residual on MRI STR = Residual > 5 × 2 × 2 mm or visible on MRI	Absence of a fundal cap was an independent predictor (on multivariate analysis) of NTR/STR (OR 2.70 (95%CI:1.68–4.36), *p* < 0.001). Other predictors of NTR/STR included older age and larger tumour size.
Fujita [10]	143	Retrosigmoid	GTR = No residual at surgery and no residual on MRI NTR = Small residual at surgery and no residual on MRI STR = Residual on MRI	Fundal cap not associated with EOR (*p* = 0.28)

*GTR* Gross total resection, *NTR* near total resection, *STR* subtotal resection, *EOR* extent of resection

### Hearing outcomes

Ten studies examined the relationship between preservation of hearing and a fundal cap (Table 4) [4, 10, 13, 15, 17, 18, 20, 22–24].

**Table 4:** Summary of studies assessing hearing outcomes

Author (Year)	Surgical Approach	*N*	Outcome
Sun [18]	Middle Fossa	138	Presence and length of fundal cap not correlated to postoperative hearing outcome.
Kosty [13]	Middle Fossa	63	Presence of fundal cap significantly associated with serviceable (*p* = 0.01) and useful (*P* = 0.02) hearing preservation.
Zhou [20]	Retrosigmoid	441	Absence of fundal cap associated with worse hearing outcomes.
Somers [4]	Retrosigmoid	26	Hearing preservation was seen in 50% of patients with fundal cap vs. 33% of those without (*p* = 0.65)
Goddard [24]	Middle Fossa	101	Postoperative serviceable hearing was seen in 60.5% of patients with fundal cap vs. 40% of those without (*p* = 0.07). Any hearing was seen in 77.6% of patients with fundal cap vs. 52% of those without (*p* = 0.01).
Mohr [22]	Retrosigmoid	63	In all patients (*n* = 106), a fundal cap was associated with hearing preservation in multivariate analysis (including tumour size and pre-op hearing). In the subgroup of patients with tumours ≤ 15 mm (*n* = 63), hearing preservation was seen in 52.8% of patients with fundal cap vs. 25.9% of those without (*p* = 0.03).
Di Maio [23]	Retrosigmoid	28	Fundal cap was associated with preservation of serviceable hearing (40% vs. 0%, *p* = 0.04)
Fujita [10]	Retrosigmoid	143	Presence of a fundal fluid cap did not significantly predict postoperative hearing loss (*p* = 0.66)
Raheja [15]	Middle Fossa	78	Fundal fluid cap did not predict hearing preservation (*p* > 0.05)
Sullivan [17]	20 Translabyrinthine + 16 Middle Fossa	36	fundal fluid distance was not correlated with postoperative PTA [Pure Tone Audiometry] r_s_=0.078 (*p* = 0.792) and SDS [Speech Discrimination Score] r_s_ = −0.056 (*p* = 0.850)

Note serviceable hearing was defined as AAO Class A or B in all studies except Fujita (2023) where hearing classification was not reported

Hearing outcomes after a middle fossa approach do not appear to be associated with a fundal cap. Three studies assessed the presence [15] and length [15, 17, 18] of the fundal cap on hearing outcomes, and found no association. In contradistinction, a smaller (*n* = 63) study demonstrated significantly greater rates of serviceable hearing in patients with a fundal cap [13].

Five studies reported on hearing outcomes following retrosigmoid craniotomy [4, 10, 20, 22, 23]. The largest of these (*n* = 441) demonstrated an almost linear correlation between the extent of CSF obliteration in the internal auditory canal and the rate of postoperative hearing loss. Patients who retained serviceable hearing postoperatively were also found to have significantly larger fundal caps compared to those who did not (*p* < 0.001) [20]. Two smaller studies reported an association between the presence of a fundal cap and hearing preservation in both large tumours (> 3 cm, *n* = 28) and small tumours (< 15 mm, *n* = 63) [22, 23]. Another study (*n* = 26) observed a trend toward better hearing outcomes in the presence of a fundal cap, though the small sample size limited statistical power. The only study that did not find a correlation between fundal cap presence and hearing preservation did not provide clear definitions for hearing outcomes, limiting interpretability [10].

**Fig. 4:**
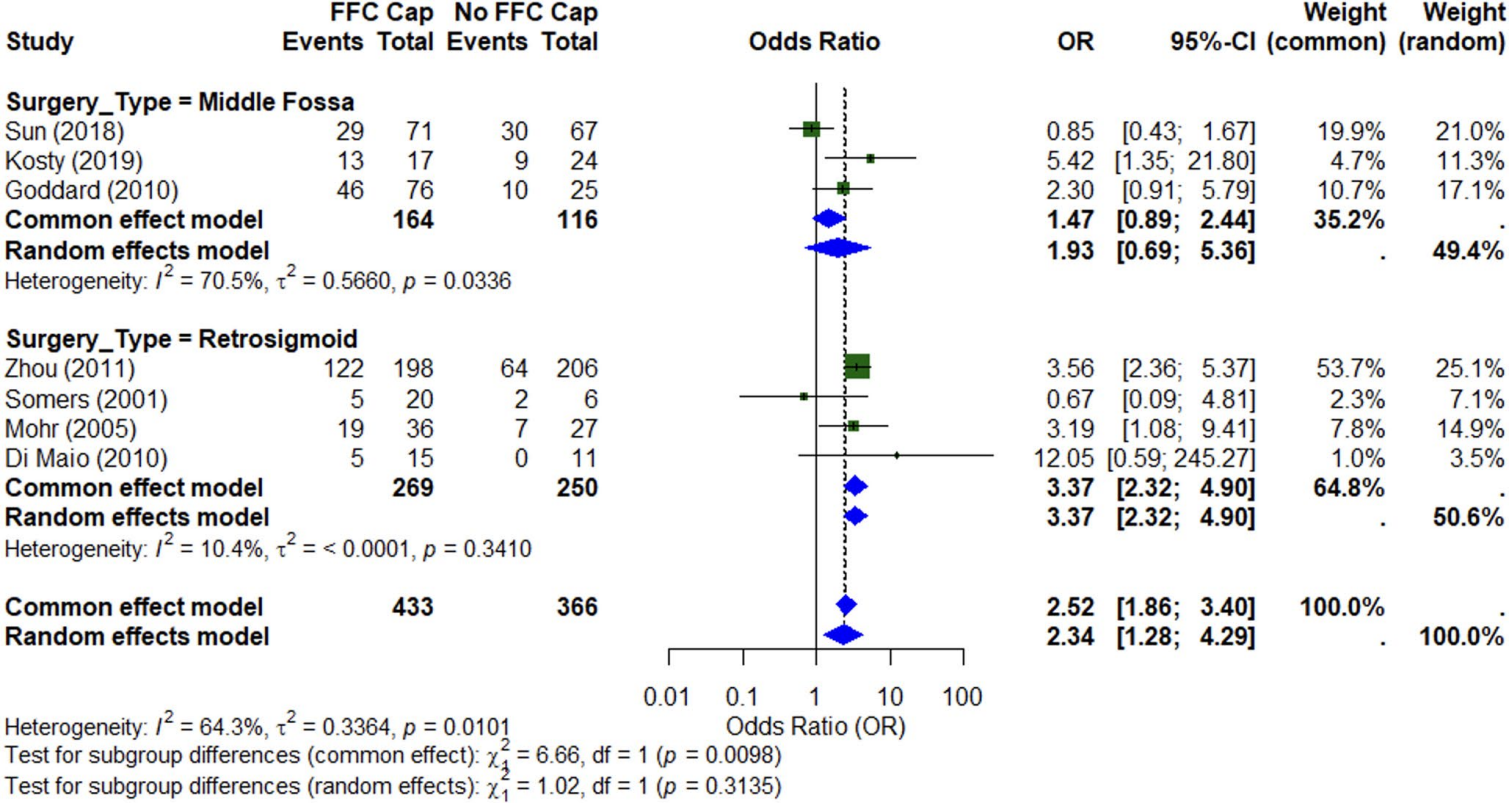
Meta-analysis of studies assessing the effect of a fundal cap on serviceable hearing outcomes after vestibular schwannoma surgery

Meta-analysis of seven studies (*n* = 785) demonstrated that the presence of a fundal CSF cap was significantly associated with improved postoperative serviceable hearing (random effects OR 2.34; 95% CI: 1.28–4.29) (Fig. 4). Subgroup analysis showed a strong and consistent effect in the retrosigmoid cohort (OR 3.37; 95% CI: 2.32–4.90; I² = 10.4%), while results in the middle fossa group were mixed and demonstrated significant heterogeneity (OR 1.47; 95% CI: 0.89–2.44; I² = 70.5%).

Two studies analysed the association of a fundal cap on hearing preservation after stereotactic radiotherapy. In one study (*n* = 106), a fundal cap nearly doubled the rate of hearing maintenance (< 20dB change in PTA) 3 years after radiosurgery (70.9% vs. 43.6%, *p* = 0.004) [19]. Similarly, in a cohort of growing vestibular schwannoma undergoing CyberKnife (*n* = 109), the rate of decreased hearing (reduction in AAO-HNS class) was significantly lower in patients with a fundal cap (HR 0.731, *p* = 0.007) [12].

No publication bias was evident on inspection of constructed funnel plots. Egger’s test for the middle fossa subgroup (t = 4.12,df = 1,*p* = 0.1516) and the retrosigmoid subgroup (t = − 0.40,df = 2,*p* = 0.7265) were not significant, indicating no substantial asymmetry in the funnel plots. Leave-one-out analysis and sensitivity analysis with studies < 30 patients removed both demonstrated no substantial change in effect.

## Discussion

A fundal CSF cap is a common and easily recognised imaging finding in vestibular schwannoma, representing a free lateral margin of the tumour arachnoid pocket. The presence of a fundal cap is associated with significantly better facial nerve outcomes after retrosigmoid craniotomy, but its consequence in translabyrinthine and middle fossa approaches is mixed. Extent of resection is greater in patients with a fundal cap, while hearing preservation is improved after retrosigmoid, but not middle fossa approaches. After radiosurgery, hearing preservation is greater in patients with a fundal cap, which may relate to lower doses delivered to the labyrinth [25].

### Fundal cap and facial nerve function

Preservation of facial nerve function is the sine qua non of vestibular schwannoma microsurgery. Previous studies have demonstrated tumour size, location relative to the long axis of the internal acoustic meatus, cystic components, and an adherent facial nerve predict facial outcomes after microsurgical resection [3, 10, 26, 27]. A fundal cap was associated with better facial nerve function after retrosigmoid craniotomy, but not after translabyrinthine or middle fossa approaches.

The facial nerve exits the brainstem anteroinferior to the vestibulocochlear nerve and rotates around it to lie in the anterosuperior quadrant of the meatus [28, 29]. The nerve thus lies superficial (anterosuperior) to the tumour during middle fossa surgery but deep to the tumour during retrosigmoid and translabyrinthine exposures. The middle fossa approach is used for small tumours with an epicentre within the meatus. Facial nerve outcomes are comparatively worse when the middle fossa is used for larger tumours that reach the cistern [30–32]. The benefit of the fundal cap - that is, a CSF pocket providing a clear tumour-arachnoid-nerve interface - is likely negated in the middle fossa exposure, as the nerve is necessarily exposed along the length of the meatus and is reliably found at the fundus anterior to the vertical crest (Bill’s bar). Similarly, the translabyrinthine approach requires exposure of the length of the meatus as well as the labyrinthine, tympanic, and mastoid segments of the facial nerve. Although the nerve lies deep to the tumour, the vertical crest serves as a constant landmark to reliably identify the nerve and begin perineural dissection. Complete exposure of the length of the meatus, coupled with a consistent landmark for facial nerve identification may explain the lack of benefit of a fundal cap on facial nerve outcome in middle fossa and translabyrinthine approaches.

During the retrosigmoid approach, the meatus is exposed through its posterior wall. The drilling is carried out sufficiently lateral to expose the lateral edge of the tumour, facilitating dissection off the facial nerve under direct vision. A fundal cap means less drilling is required, and the nerve can be found easily on the anterior superior aspect of the meatus after sectioning the vestibular nerves. Extent of meatal exposure may be limited laterally by the posterior semicircular canal, crus commune, and vestibule, and inferiorly by a high riding jugular bulb. In these cases, a fundal cap increases the likelihood that the lateral aspect of the tumour is medial to the labyrinth, obviating the need for blind dissection in the meatus. These benefits likely underlie the improved facial nerve outcomes seen with a fundal cap in retrosigmoid approaches.

### Fundal cap and extent of resection

Surgical philosophy for vestibular schwannoma varies between surgeons, where extent of resection and oncologic control must be balanced against cranial nerve preservation. Quality of life is related to facial nerve function and extent of resection, but the differences between treatment modalities is minimal [33–35]. Complete resection relieves anxiety related to residual tumour growth and reduces the risk of recurrence, but may be associated with an increased risk of facial nerve injury [36, 37]. The presence of a fundal cap was associated with a two-fold increase in the rate of gross total resection in this meta-analysis including retrosigmoid and translabyrinthine approaches. The fundus of the IAM is the most common location for unintentional residual tumour, and residual within the IAM is an independent risk factor for tumour progression after subtotal resection [38, 39]. A fundal cap facilitates visualisation of the lateral edge of the tumour, reducing the chance of unintentional residual in the meatus. Similarly, it facilitates a clear plane to begin lateral to medial perineural dissection from the facial nerve, improving resection rates.

### Fundal cap and hearing preservation

Hearing dysfunction in vestibular schwannoma is progressive, and may relate to stretch and microvascular dysfunction of the cochlear nerve, endolymphatic hydrodynamic dysfunction, compartment syndrome within the meatal fundus, and the peritumoral inflammatory milieu [40, 41]. Intracanalicular pressure correlates with hearing function preoperatively, and a greater volume of tumour within the meatus (smaller fundal cap) is associated with worse preoperative hearing and higher intracanicular pressures [20, 42, 43], although a confounding effect of the pressure monitor itself on intracannicular pressure can not be excluded. A larger fundal cap is protective against hearing deterioration, potentially acting as a buffer against the development of a meatal compartment syndrome [44].

A fundal cap was associated with hearing preservation after retrosigmoid craniotomy, but not middle fossa approaches. Similar to facial nerve preservation, the fundal cap provides a clear lateral start to the perineurial plane of dissection of the cochlear nerve. It also means less lateral meatal drilling is required, reducing the risk of inadvertent violation of the otic capsule. No significant difference is seen after middle fossa approaches, likely attributable to the extensive exposure of the nerve along the length of the meatus, lower risk of inadvertent otic capsule injury, and its association with smaller tumours [15].

### Limitations

Our study has several limitations. Included studies were retrospective with small to moderate sample sizes. Data on preoperative hearing and facial nerve function was inconsistently recorded, and outcomes were not uniformly stratified by surgical approach or tumour size, limiting meta-analysis power. There is inherent heterogeneity in surgical technique and surgeon aggressiveness, with consequences for facial nerve preservation and EOR. Additionally, surgical cohorts may capture the learning curve of the surgeons and it remains to be seen whether surgeon experience can minimise or negate the prognostic effect of the fundal cap.

EOR measurement was not standardised across studies. Moreover, the two studies that analysed the affect of the fundal cap on EOR included a mix of retrosigmoid and translabyrthine approaches. Fundal cap size correlated with larger tumour size, confounding and potentially artificially diminishing its effects on EOR [18]. Overall, it would appear that there is a signal for improved EOR in the presence of a fundal cap, however further studies are required to confirm this.

Variations in MRI acquisition parameters may impact the detection of a fundal cap, including slice thickness, pulse sequences, field strength, and T2 weighting. There is no universally accepted MRI protocol or sequence for defining a fundal cap. Heavily T2 weighted fast spin echo sequences may be more sensitive than gradient echo sequences due to the magnetic susceptibility artefacts of the latter.

The definition and measurement of the fundal cap was not standardised; measurement techniques varied, and inter-observer agreement was not reported. No study compared linear measurement to binary (presence vs. absence) definitions. Larger fundal caps are associated with better outcomes in some studies, but no threshold distance for benefit has been established [20]. In line with the majority of the current data, we suggest an interim definition of a fundal cap as a ≥1 mm length of signal in the fundus of the internal acoustic meatus, lateral to the enhancing tumour, that follows CSF intensity on all sequences.

Future research should focus on establishing a more rigorous definition that maximises interobserver reliability and prognostic significance. Further anatomical delineation of the relationship of the arachnoid, perineurium, tumour, and cranial nerves within the fundal cap would provide insights to guide surgical technique.

## Conclusion

The fundal CSF cap is an important predictor of improved facial nerve function, and hearing preservation after retrosigmoid craniotomy for vestibular schwannoma, and a signal for improved extent of resection that requires further investigation. Its importance in middle fossa and translabyrinthine surgery is less clear, which reflects the anatomical considerations of each approach. The presence or absence of a fundal cap should be documented preoperatively and used to guide more nuanced risk assessment for preoperative patient counselling. Future studies should endeavour to establish a consistent definition of a fundal cap to unify prospective studies.

## Data Availability

The data analyzed in this systematic review are publicly available in the original articles listed in the references section.
